# Support for art interventions in children's hospitals and design strategies for alleviating negative emotions

**DOI:** 10.3389/fped.2025.1649799

**Published:** 2026-01-12

**Authors:** Lianyu Wang, Dong Zheng

**Affiliations:** 1 School of Fine Arts, Sichuan University of Science and Engineering, Zigong, China; 2 School of Arts and Design, Guangzhou University, Guangzhou, China

**Keywords:** art intervention, children's hospitals, emotions, healing needs, medical space design

## Abstract

**Issue:**

Children experience heightened negative emotions during healthcare visits. Art represents an effective non-pharmacological intervention, but its potential remains underutilized in Chinese children's hospitals.

**Objective:**

To alleviate children's negative emotions, enhance the artistic quality of hospital spaces, and support the development of humanized, child-friendly medical environments.

**Methods:**

A mixed-methods approach was employed to investigate the spatial environments of two children's hospitals in Shanghai, China. Pediatric patients aged 3–14 years and their parents were studied using on-site surveys, questionnaire investigations, and naturalistic observation.

**Results:**

Support for art intervention was reported by 95.89% of children and 97.75% of parents. Existing artworks showed limited effectiveness, with 20.55% of children not noticing them and only 9.59% rating them as highly aesthetic. The blood-drawing and infusion areas elicited the strongest negative emotional responses, whereas the courtyard space was associated with the lowest levels of negative affect. For art preferences and activity demands in the infusion area, 193 valid questionnaires were collected. Analysis identified four Must-be (M) attributes, three One-dimensional (O) attributes, five Attractive (A) attributes, three Indifferent (I) attributes, and one Reverse (R) attribute, with no dubious attributes detected.

**Conclusion:**

This study provides empirical support for integrating art into children's hospital environments and proposes targeted art design strategies for pediatric infusion areas. The findings offer practical guidance for improving children's medical experiences and advancing the development of child-friendly healthcare spaces.

## 1 Introduction

Individuals exhibit varied emotional responses across environments. Research indicates that approximately 20% of individuals experience “white coat syndrome,” a fear response commonly observed in medical settings. This reaction represents a typical physiological phenomenon, as hospitals are widely associated with illness and injury (1).

Children, as a distinct population, show a higher propensity for negative emotions, including anxiety and resistance, when encountering healthcare professionals in white attire (2). Extensive evidence from healing-oriented research has demonstrated that alleviating negative emotions in pediatric patients improves treatment processes and recovery outcomes (3–7). An emotional transmission mechanism also exists among pediatric patients, their families, and medical staff, with the emotional state of the child forming the core of this transmission loop. Regulating children's emotions during medical treatment therefore plays a critical role in fostering harmonious doctor–patient relationships (8). To reduce negative emotions experienced by children during medical care, a range of interventions has been introduced, including play therapy and storytelling (9), clown visits (10), drawing art therapy (11), and doctor cross-dressing (12). Art, as a non-pharmacological intervention, has been shown to significantly enhance mental well-being (13–17). At the policy level, the United States enacted the “Percent for Art” Act in the 1960s. This policy requires that 1% of the total construction cost of new public buildings, including hospitals, be allocated to art procurement, with artworks displayed in publicly accessible areas to improve environmental quality through artistic means (18, 19). Contemporary hospital art encompasses a wide range of forms, including murals, sculptures, art installations, digital art (such as projection-based works and interactive digital displays), music, and on-site cinemas, which are commonly located in public waiting areas (20). Many countries and healthcare institutions are increasingly incorporating artworks and installations to enhance the aesthetic quality of medical environments and to support patients' emotional well-being (21). This trend highlights the positive role of art interventions in hospital settings. Empirical studies have shown that the application of art in hospitals significantly improves patient satisfaction with medical institutions (22–24). In addition, various art-based therapeutic activities have demonstrated positive emotional effects on pediatric patients and have been associated with increased compliance during treatment and medical examinations (25, 26). Researchers have also translated and validated the Italian version of the Arts Observation Scale (ArtsObS), contributing to the establishment of standardized tools for evaluating the effectiveness of art therapy in pediatric healthcare contexts (27). Although existing studies have examined artworks and therapeutic activities from multiple perspectives (28–31) and explored optimized design strategies for specialized children's hospitals (32), limited attention has been given to the targeted integration of art and therapeutic activities across different hospital spaces to better address children's specific emotional needs.

On this basis, the present study addresses the following three research questions from the perspectives of children and their parents:

1. What levels of support do children and parents express toward art interventions in pediatric hospitals?
2. What emotional states do pediatric patients experience in different hospital settings, and what behavioral interventions are employed to alleviate these emotions?
3. What art preferences and requirements emerge in environments associated with heightened negative emotions, and how can targeted art intervention strategies be developed for these contexts?

By examining these questions, the study seeks to strengthen the theoretical framework of art interventions in children's hospitals, provide evidence-based guidance for improving pediatric medical environments, and promote the integration of spatial design and art to enhance children's overall healthcare experiences.

## 2 Research framework

**Figure 1:**
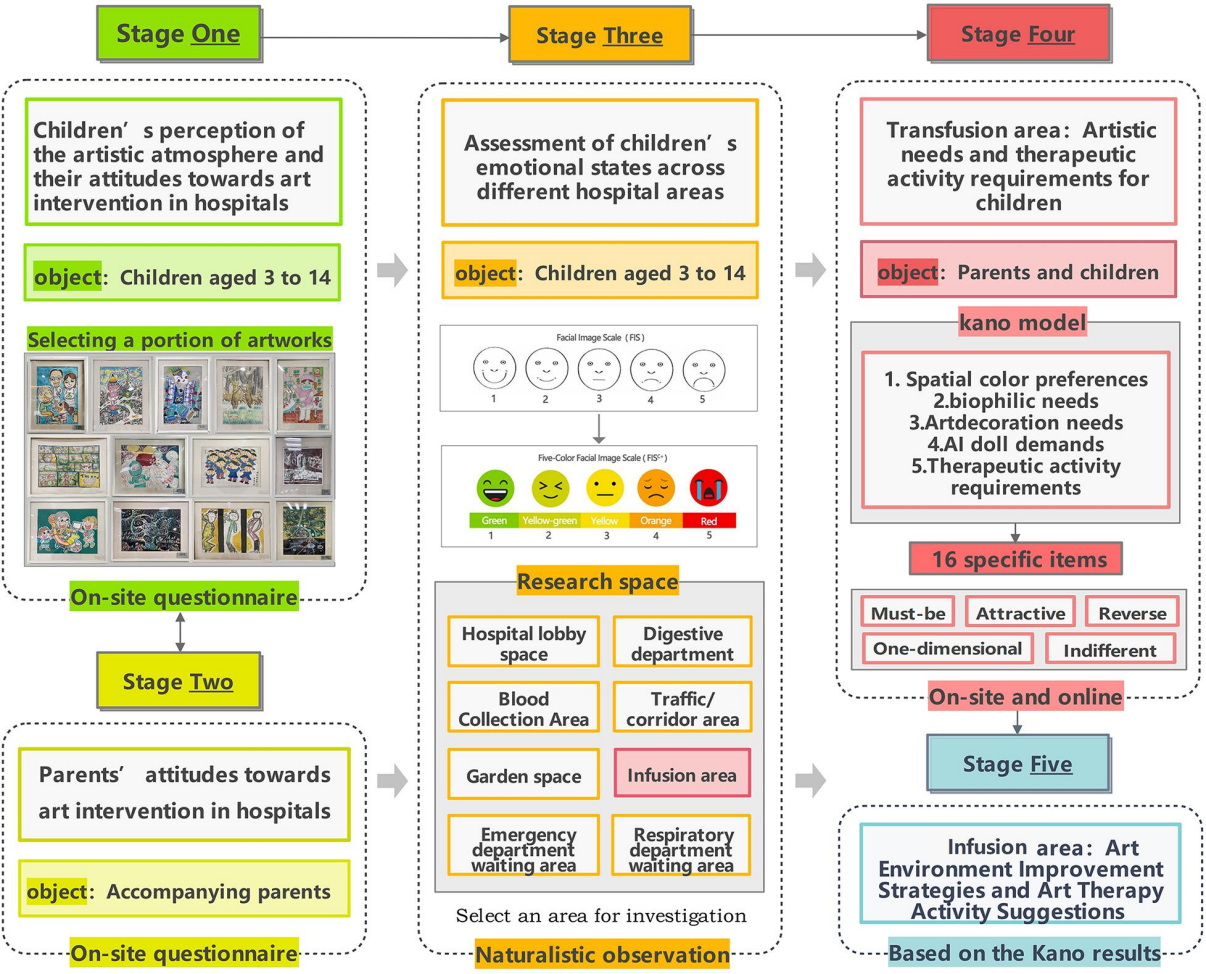
Research framework.

This study selected two children's hospitals in Shanghai as field investigation sites: the Luding Road Branch of Shanghai Children's Hospital and the Children's Hospital Affiliated with Fudan University. The former is the earliest children's hospital on the Chinese mainland, while the latter is among the first group of national children's medical centers. Data collection was conducted in late March and early April. On-site investigations and questionnaire distribution were carried out at each hospital during both morning and afternoon periods. The study was organized into five stages, as illustrated in Figure 1.

## 3 Methods

This study adopted a multimethod approach. Within the research framework described in the previous section, data collection in the first, second, and fourth phases was conducted using questionnaire surveys, while the third phase employed a combined approach involving natural observation and field investigation.

### 3.1 Survey areas and screening criteria

The survey areas for assessing children's emotional states included the hospital lobby, emergency waiting area, respiratory department waiting area, gastroenterology department waiting area, blood-drawing area, infusion area, main corridor spaces, and courtyard spaces, yielding a total of eight distinct locations. The respiratory and gastroenterology waiting areas were selected as key observation points based on a 2020 survey conducted across 11 major cities in China, which identified respiratory infections and diarrhea as the two most prevalent pediatric diseases (33). These departments experience high patient volumes and frequent visits, making their waiting areas particularly suitable for investigation.

Participant selection followed defined criteria. Children with severe acute or chronic illnesses, those in markedly poor emotional states, those with cognitive or language impairments, and those unable to cooperate were excluded to avoid interference with health status or rest. Given the limited language comprehension and expressive abilities of younger children, parental assistance was required for questionnaire completion. Families were excluded if parents were unable to adequately assist due to urgent work demands, such as frequent work-related phone calls, or personal matters.

### 3.2 Survey methods for children's attitudes toward art

During this phase, on-site questionnaires were administered to examine children's perceptions of the hospital's artistic environment and their level of support for the incorporation of art into hospital spaces.

#### 3.2.1 Sampling method

The sample was selected using a combination of stratified and random sampling methods. Stratification was conducted across three dimensions: age, gender, and hospital area. Participants were grouped into three age ranges (3–6 years, 7–9 years, and 10–14 years), with an approximately equal gender ratio maintained within each group. In most Chinese hospitals, 14 years is defined as the upper age limit for pediatric care, as children below this age are considered physiologically and psychologically immature and therefore require pediatric expertise. Random sampling was then applied within each stratum to ensure representative coverage across all categories.

#### 3.2.2 Questionnaire design

The questionnaire consisted of five concise items. The first two items collected information on gender and age. The third item asked whether children had noticed wall paintings or other artworks within the hospital. The fourth item assessed children's aesthetic evaluations of wall paintings and displayed artworks, with sample images from the study hospitals provided as references. The fifth item asked whether children wished to see more artworks in hospital environments.

### 3.3 Survey methods for parents' attitudes toward art

For the parent component, on-site questionnaires were similarly distributed to assess parents' support for integrating art into hospital spaces during their children's medical treatment. Sampling followed the same combined stratified and random approach, and descriptive statistical analysis was applied to the collected data.

The parent questionnaire included three attitudinal items: (1) whether the artwork positively influenced the child's mood during hospital visits; (2) whether additional artworks or healing activities in pediatric healthcare facilities were supported; and (3) whether the creation of designated art exhibition spaces within children's hospitals was supported.

### 3.4 Methods of assessing children's emotional status

**Figure 2:**
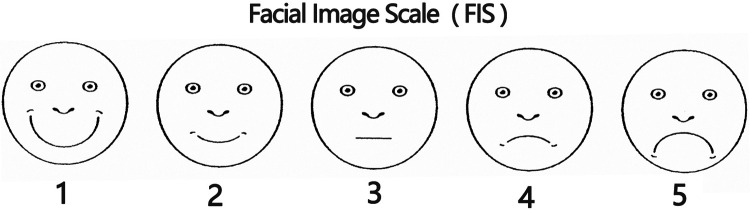
Facial image scale (FIS).

**Figure 3:**
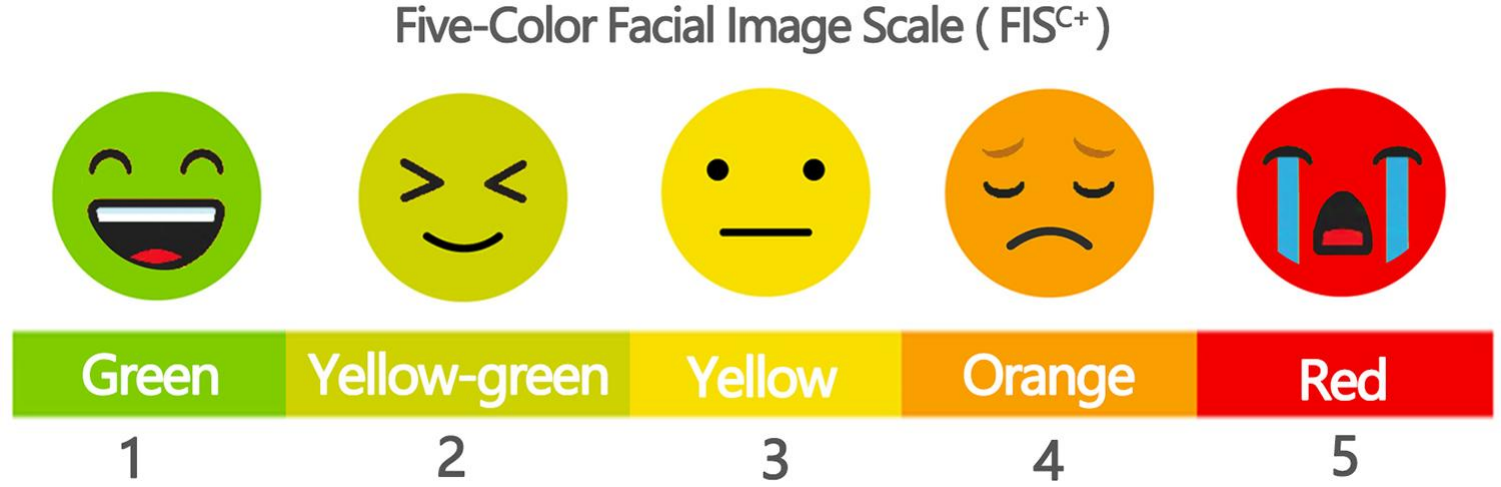
Five-color facial image scale (FIS^C+^).

This section primarily employed natural observation and applied the Facial Image Scale (FIS) (34) to evaluate children's emotional states within the selected survey areas, as shown in Figure 2. The scale classifies children's emotional status into five levels. Building on the original FIS, color coding was incorporated to improve visual clarity and intuitiveness, and the revised scale was designated the Five-Color Facial Image Scale (FIS^C+^). Specifically, red (5 points) indicates a poor emotional state accompanied by crying; orange (4 points) denotes low spirits, with observable signs of displeasure or irritability at the verbal or behavioral level; yellow (3 points) represents a calm state; yellow-green (2 points) indicates a good mood; and green (1 point) signifies a pleasant smile or observable signs of happiness in language or behavior, as illustrated in Figure 3. To ensure consistency and comparability across observations, a single observer was assigned, and the number of children assessed in each survey area was standardized at 100.

### 3.5 Survey methods for parents' and children's art preferences in the infusion area

During this phase, both on-site and online questionnaires were administered to examine parents' and children's artistic preferences in infusion areas, as well as their preferences for therapeutic activities. Offline respondents consisted of children and their parents present in the infusion areas of the two hospitals, while online respondents included children and parents with prior infusion experience in children's hospitals.

**Figure 4:**
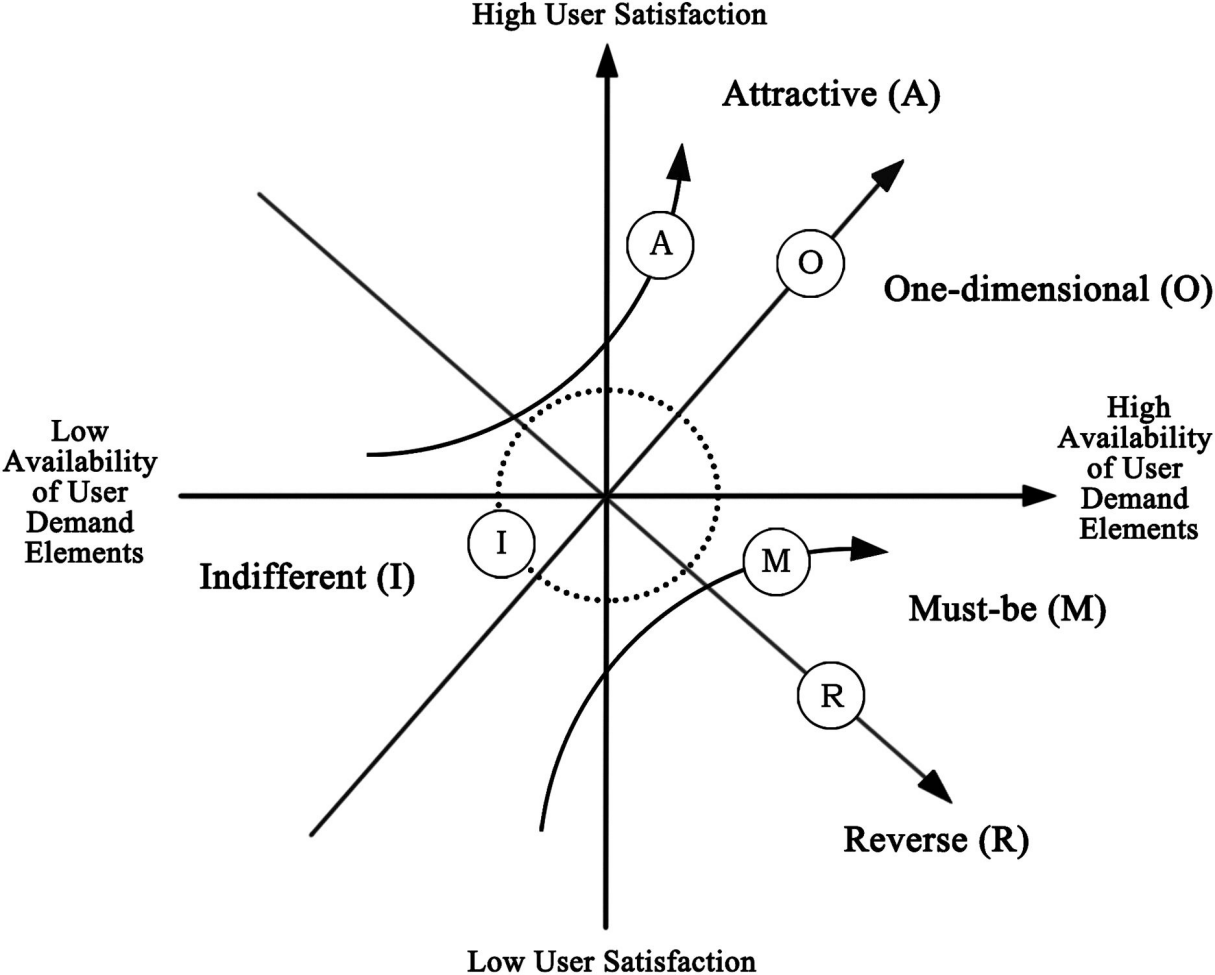
Relationship between demand satisfaction and user satisfaction in the Kano model.

This section adopted the Kano questionnaire. The Kano model is a method for classifying and prioritizing user needs (35) and is widely applied in requirement analysis and product improvement studies. The model involves collecting users' responses to different functional attributes and analyzing their associated satisfaction levels (36). User needs are categorized into five types: Must-be (M), Attractive (A), One-dimensional (O), Reverse (R), and Indifferent (I). Must-be needs represent fundamental requirements and form the baseline of satisfaction. Attractive needs are not essential but can markedly increase satisfaction when present and do not reduce satisfaction when absent. One-dimensional needs show a direct proportional relationship with satisfaction, with higher fulfillment leading to higher satisfaction and vice versa. Reverse needs can generate dissatisfaction when present and may increase satisfaction when absent. Indifferent needs exert little influence on satisfaction regardless of whether they are fulfilled**.** The relationship between need fulfillment and user satisfaction is illustrated in Figure 4.

## 4 Research results

### 4.1 Children's attitudes toward art intervention in hospitals

**Figure 5:**
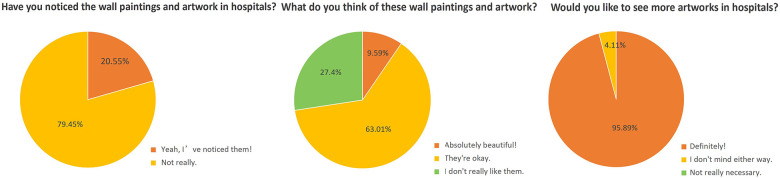
Pie chart of data distribution for the children's group.

A total of 100 questionnaires were distributed; 73 valid responses were ultimately obtained. The sample included 41 boys and 32 girls. In terms of age, 38 children were aged 3–6 years, 27 were aged 7–9 years, and 8 were aged 10–14 years. The statistical results for Questions 3–5 are presented in Figure 5.

The main findings are as follows. First, 20.55% of the children reported not noticing murals or paintings in the hospital. Only 9.59% rated the artworks as having high aesthetic value, whereas 63.01% considered the aesthetic quality to be average. These results suggest that both the artistic atmosphere and the quality of artworks in the surveyed hospitals warrant further improvement. Second, Pearson's chi-square analysis showed no significant association between gender and the observation of paintings or artworks (*χ*² = 2.26, df = 1, *P* = 0.133 > 0.05). Third, 95.89% of the surveyed children expressed support for the introduction of artworks into the hospital environment.

### 4.2 Parents' attitudes toward art intervention in hospitals

**Figure 6:**
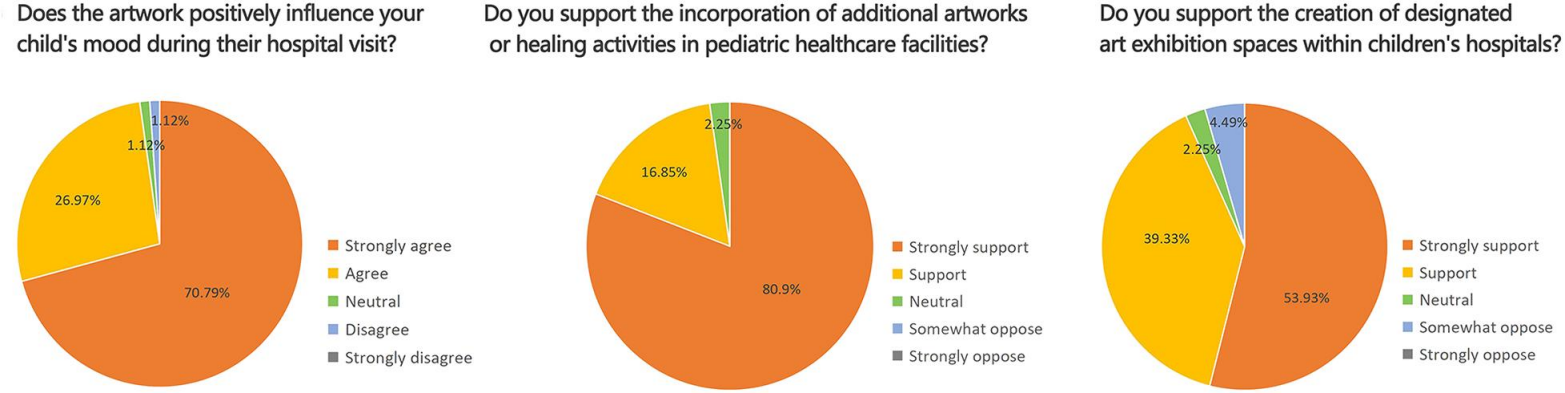
Pie chart of data distribution for the parents' group.

A total of 100 questionnaires were distributed to parents, of which 89 valid responses were collected on site. The data distribution is shown in Figure 6.

The results indicate that most parents strongly acknowledged the positive role of artworks in alleviating children's anxiety during medical treatment. Only one parent reported a neutral attitude, and one parent expressed disagreement. Regarding the incorporation of artworks and art-based therapeutic activities in hospitals, 97.75% of parents indicated support. Support for establishing dedicated art exhibition areas in hospitals was slightly lower, with 93.26% of parents reporting strong support or support, suggesting that a small proportion of parents held reservations. Overall, the findings demonstrate that parents generally maintain positive and supportive attitudes toward the integration of art into children's hospitals.

### 4.3 Assessment of children's emotional status

Higher numerical values correspond to more negative emotional states among pediatric patients in the respective areas. The blood-drawing area (383 points), infusion area (380 points), and emergency waiting area (363 points) exhibited the highest emotional scores, indicating more pronounced negative emotions. By contrast, the hospital lobby, respiratory department waiting area, and gastroenterology department waiting area showed emotional scores between 300 and 350 points, reflecting moderate emotional states. The main corridor area (291 points) and courtyard space (242 points) recorded scores lower than 300 points, indicating relatively more positive emotional states among pediatric patients.

During the assessment of children's emotional states, parents were observed employing a range of spontaneous coping strategies to manage their children's negative emotions within the hospital environment. The most common soothing approach involved playing cartoons on electronic devices to distract children and reduce anxiety. Parents also adopted supplementary strategies, including bringing balloons, drawing boards, toys, snacks, and other items; guiding children to observe outdoor scenery through windows; and providing homework or learning materials for older children. These behaviors demonstrate parents' active adaptation to the stress associated with children's medical treatment and their attentiveness to children's psychological needs.

**Figure 7:**
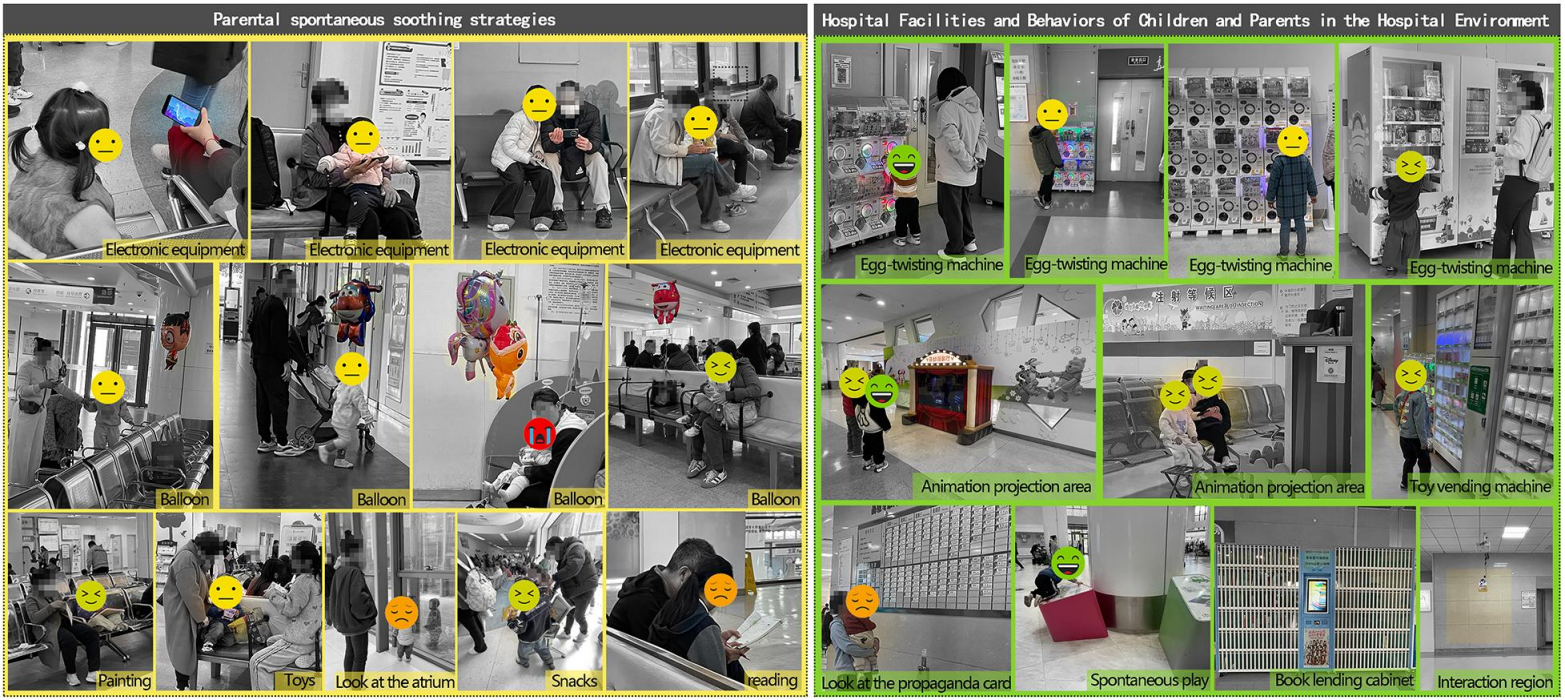
Photographs of parental soothing strategies and child-oriented hospital facilities.

The surveyed hospitals have also installed various child-oriented facilities tailored to pediatric characteristics. In both hospitals, main corridor areas were generally equipped with capsule toy machines and toy vending machines, along with cartoon playback devices. One hospital placed cartoon playback equipment in the infusion waiting area, while the other located it in the main waiting hall. The Children's Hospital Affiliated with Fudan University was further equipped with a book-borrowing cabinet and an interactive projection game area. On-site observations showed that the book-borrowing cabinet contained only a limited number of books, with no observed borrowing behavior, and the interactive projection game area was frequently closed. Despite these limitations, children were found to engage spontaneously with the hospital environment for play, demonstrating notable adaptability and creativity. For example, in the lobby of the Luding Road Branch of Shanghai Children's Hospital, children were observed climbing and playing on staggered floor-level signage. Representative photographs recorded during the survey are presented in Figure 7.

Survey findings can be summarized as follows:

The blood collection area and infusion area are the locations where pediatric patients exhibit the most intense negative emotions, while the courtyard and main corridor areas show comparatively more positive emotional states and represent the most favorable environments in terms of emotional status.
The primary items parents bring to alleviate children's negative emotions during medical treatment include electronic devices for playing cartoons or video games, balloons, drawing boards, toys, snacks, and similar objects, mainly to divert attention or reduce anxiety.
Children's hospitals have installed facilities such as capsule toy machines, toy vending machines, cartoon playback devices, book-borrowing cabinets, and interactive projection game areas to address pediatric needs, reflecting an effort to reduce medical anxiety and improve children's healthcare experiences through non-medical interventions.

### 4.4 Artistic needs and healing activity demands in the infusion area

Based on the preceding survey results and the observation that children in infusion areas remain relatively stationary for extended periods, this area and its surrounding spaces present strong potential for artistic practice and healing activities within hospitals. On this basis, Task Four implemented a targeted questionnaire survey in the infusion area to examine the spatial and activity-related needs and preferences of children and their parents.

#### 4.4.1 Extraction of needs items in the infusion area

To improve questionnaire structure and systematically extract user requirements, one university faculty member with relevant expertise and two doctoral researchers were invited to synthesize and refine core demands using the KJ method. The finalized questionnaire subdivided artistic and healing activity needs in pediatric infusion areas into 16 items across five dimensions: spatial color preferences, art decoration preferences, healing activity preferences, biophilic preferences, and AI toy preferences, as shown in Table 1. A sample of the Kano model questionnaire form is presented in Table 2.

**Table 1:** Classification and description of art and healing activity needs in the infusion area.

Requirements	Code	Description
Warm color scheme	A1	The space uses war m colors such as orange and yellow
Cool color scheme	A2	The space uses cool colors such as blue and green
Neutral color scheme	A3	The space is primarily in neutral colors such as white and gray
Wall Murals	A4	The infusion area's walls are decorated with cartoon patterns or beautiful drawings
Art Paintings	A5	The infusion area's walls display art pieces
Static Art Installations	A6	The infusion area features static art installations with aesthetic value
Interactive Art Installations	A7	The infusion area includes interactive art installations that can engage children in fun interactions
Drawing Area	A8	A designated drawing area in the infusion area with pens,paper,and other tools provided
Handicraft Area	A9	A handicraft area in the infusion area where materials and tools for making crafts can be purchased
Reading Corner	A10	A reading corner in the infusion area with books suitable for children
Board Game Area	A11	A board game area in the infusion area with a variety of games provided
Movie-Watching Area	A12	A viewing area in the infusion area that plays health education videos or cartoons
Nature-themed Wall Projections	A13	The infusion area's walls project nature-themed images, such as forests, oceans
Plant Corner	A14	A plant corner in the infusion area(with artificial plants, greenery boxes,or aquariums)
Sunlight area (sunroom)	A15	A specific area in the infusion area is designed to create a sunroom effect through special design
AI Interactive Dolls	A16	The infusion area features AI interactive dolls with intelligent interaction capabilities

**Table 2:** Kano model questionnaire form.

6. Attitude towards static art installations/sculptures	
Static art sculptures are placed	Static art sculptures are not placed
l like it l expect it l am neutral l can tolerate it l dislike it	l like it l expect it l am neutral l can tolerate it l dislike it

An initial on-site survey was conducted in the infusion area, during which 100 questionnaires were distributed and 71 valid responses were collected. Subsequently, 150 questionnaires were distributed online via the WJX platform. After excluding responses with evident logical inconsistencies or unrealistically short completion times, 122 valid online questionnaires were retained, corresponding to an online response rate of 81.33%. After data integration, a total of 193 valid questionnaires were obtained.

#### 4.4.2 Reliability and validity testing

Questionnaire reliability and validity were evaluated using SPSS software. Cronbach's α coefficients for the overall questionnaire, positive items, and negative items were 0.738, 0.956, and 0.963, respectively, indicating satisfactory internal consistency. Validity testing yielded a Kaiser-Meyer-Olkin (KMO) value of 0.963, and Bartlett's test of sphericity produced a *p*-value below 0.05. These results confirm that the questionnaire demonstrates high reliability and validity.

#### 4.4.3 Results analysis

The analysis results were compared with the Kano model classification table, as shown in Table 3. By statistically analyzing responses to the positive and negative items, the Kano attribute corresponding to the highest proportion was identified for each need. The results indicated four Must-be (M) attributes, three One-dimensional (O) attributes, five Attractive (A) attributes, three Indifferent (I) attributes, and one Reverse (R) attribute. No Questionable (Q) attributes were identified. Detailed classification results are presented in Table 4.

**Table 3:** Comparison of KANO model evaluation results.

Participant attitude towards needs		Negative questions				
		Like it	Expect it	Neutral	Tolerable it	Dislike it
Positive Questions	Like it	Q	A	A	A	O
	Expect it	R	I	I	I	M
	Neutral	R	I	I	I	M
	Tolerable it	R	I	I	I	M
	Dislike it	R	R	R	R	Q

A, Attractive; O, One-dimensional; M, Must-be, I, Indifferent; R, Reverse; Q, Questionable.

**Table 4:** Attribute classification analysis of art and healing activity needs in the infusion area.

Code	A	O	M	I	R	Q	Type	Worse	Better
A1	31	19	26	108	9	0	I	−24.46%	27.17%
A2	28	21	73	63	8	0	M	−50.81%	26.49%
A3	32	28	19	18	96	0	R	−48.45%	61.86%
A4	33	23	91	35	11	0	M	−62.64%	30.77%
A5	51	80	17	32	13	0	O	−53.89%	72.78%
A6	53	18	26	84	12	0	I	−24.31%	39.23%
A7	98	20	18	45	12	0	A	−20.99%	65.19%
A8	31	63	42	45	12	0	O	−58.01%	51.93%
A9	97	21	26	41	8	0	A	−25.41%	63.78%
A10	33	19	95	35	11	0	M	−62.64%	28.57%
A11	51	17	18	95	12	0	I	−19.34%	37.57%
A12	27	22	93	38	13	0	M	−63.89%	27.22%
A13	113	15	19	37	9	0	A	−18.48%	69.57%
A14	39	60	45	36	13	0	O	−58.33%	55.00%
A15	107	25	18	34	9	0	A	−23.37%	71.74%
A16	108	15	17	47	6	0	A	−17.11%	65.78%

A, Attractive; O, One-dimensional; M, Must-be, I, Indifferent; R, Reverse; Q, Questionable.

Next, to further examine the relationship between need fulfillment and satisfaction among children and their parents in the infusion area, the Better–Worse coefficient analysis method was applied. The Better coefficient (1) and the Worse coefficient (2) were calculated using the following formula (37):

$$\mathrm{Better}=\frac{A+O}{A+O+M+I}$$ (1)

$$\mathrm{Worse}=\frac{O+M}{(A+O+M+I)\times (-1)}$$ (2)

**Figure 8:**
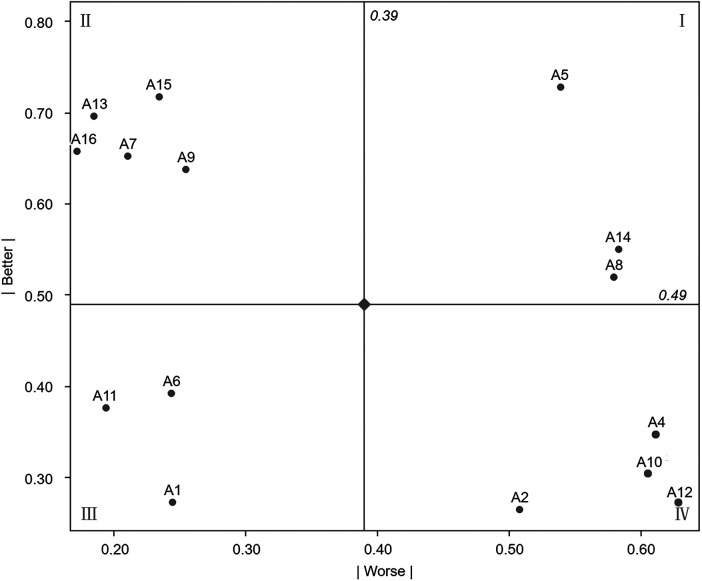
Better-worse four-quadrant scatter diagram.

The Better-Worse coefficients for each need item, excluding reverse needs, were calculated and plotted on a four-quadrant scatter diagram, as shown in Figure 8.

Quadrant IV (Must-be needs) is characterized by low Better values and high Worse values and includes A12, A4, A2, and A10. Must-be needs represent the most basic requirements. Meeting these needs in the infusion area does not markedly increase satisfaction among children and parents, whereas failure to meet them leads to a substantial decline in satisfaction.

Quadrant I (One-dimensional needs) is characterized by high Better values and high Worse values and includes A14, A5, and A8. Needs in this quadrant exhibit a direct relationship with user satisfaction. Higher levels of fulfillment result in increased satisfaction among children and parents, while lower levels lead to decreased satisfaction.

Quadrant II (Attractive needs) is characterized by high Better values and low Worse values and includes A16, A13, A9, A15, and A7. Although not essential, fulfillment of these needs can generate additional pleasure for children and parents in the infusion area, while their absence does not negatively affect satisfaction. These needs may be selectively addressed according to the available resources of individual hospitals.

Quadrant III (Indifferent needs) is characterized by low Better values and similarly low Worse values and includes A6, A11, and A1. These needs exert minimal influence on satisfaction regardless of whether they are fulfilled.

To further distinguish the relative importance of individual need items within the same attribute category and to determine priority levels among different need attributes in the pediatric infusion area, secondary ranking was performed by calculating sensitivity values. A higher sensitivity value indicates greater importance and a higher priority within the same attribute category.

The calculation formula is as follows:

$$R=\sqrt{{\left|\mathrm{Better}\right|}^{2}+{\left|\mathrm{Worse}\right|}^{2}}$$

Based on the calculation results, the priority ranking of the four categories of needs and healing activities in the pediatric infusion area is as follows:

Must-be (M) needs: Wall murals (A4) > Movie-Watching Area (A12) > Reading corner (A10) > Cool color scheme (A2).
One-dimensional (O) needs: Art paintings (A5) > Plant corner (A14) > Drawing area (A8).
Attractive (A) needs: Sunroom (A15) > Nature-themed images (A13) > Handicraft activity area (A9) > Interactive art installations (A7) > AI toys (A16).
Indifferent (I) needs: Static art installations (A6) > Board game activity area (A11) > Warm color scheme (A1).
Healing activities: Drawing area (A8) > Movie-Watching Area (A12) > Reading corner (A10) > Handicraft activity area (A9) > Board game activity area (A11).

**Figure 9:**
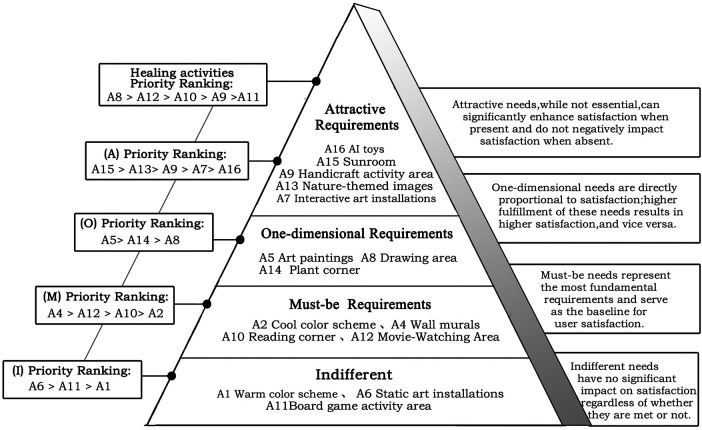
Art and healing needs model diagram for the infusion area.

This ranking provides a structured reference for shaping the artistic atmosphere and planning healing activities in pediatric infusion areas. The artistic needs model for the infusion area is illustrated in Figure 9.

## 5 Discussion

The on-site survey process proved more challenging than expected, largely due to anxiety and resistance among child participants and their parents, which resulted in a relatively low questionnaire response rate. Despite these constraints, the findings remain informative and support several clear conclusions.

Both children and parents expressed favorable attitudes toward the introduction of art-based interventions in children's hospitals and acknowledged their role in alleviating negative emotions during medical treatment. These findings are consistent with previous studies that emphasize the value of art interventions in healthcare environments (38, 39). The survey also revealed shortcomings in current art intervention practices. A notable proportion of children (20.55%) did not notice murals or artworks in the surveyed hospitals, and 63.01% rated the aesthetic quality of existing artworks as average. These results indicate substantial potential for improvement in the construction of hospital art environments, particularly in enhancing the visibility and aesthetic appeal of artworks.

The blood-drawing area and infusion area exhibited significantly higher levels of negative emotion than other hospital spaces, identifying them as high-stress environments during pediatric medical visits. In contrast, the main corridor area and courtyard space were associated with more positive emotional states, suggesting that exposure to natural elements and open spaces contributes to the mitigation of negative emotions in children (40, 41).

Observational data showed that parents most frequently relied on playing cartoons on electronic devices as a soothing strategy. Capsule toy machines located in hospital corridors also demonstrated a degree of positive distractive effect by attracting children's attention. Notably, during the nearly half-month observation period, no children were observed stopping to view wall paintings. This finding indicates that, despite the physical presence of murals and paintings, their emotional impact was limited. The result points to deficiencies in the current application of positive distraction through art and underscores the need for more effective and engaging art intervention strategies.

To address the unfavorable emotional states observed in infusion areas, further investigation into artistic preferences and activity demands was conducted. Among the 16 surveyed items, four were classified as Must-be (M) needs, three as One-dimensional (O) needs, five as Attractive (A) needs, three as Indifferent (I) needs, and one as a Reverse (R) need. According to the Kano model, the satisfaction hierarchy of demand attributes follows the order M > O > A > I. The subsequent discussion therefore focuses on art design strategies and environmental improvement recommendations informed by this hierarchy.

To begin with, Must-be needs should be addressed as a priority. These include essential wall decorations (A4), the establishment of a movie-watching area (A12), and a reading corner (A10). The movie-watching area should provide animated films or health- and medical-science popularization videos. In addition, the infusion area should adopt a cool color scheme (A2) as the dominant spatial tone. In children's hospital environments, green and blue are widely recognized for their calming and restorative effects (42). After Must-be needs are satisfied, the design of the infusion area should focus on fulfilling One-dimensional needs, which function as key determinants of user satisfaction. Art paintings (A5), identified as the most important One-dimensional need, reflect recognition by both parents and children of the emotional pleasure derived from viewing high-quality artworks. Previous studies have demonstrated that artworks displayed in hospital environments can improve patients' emotional states (43). A designated drawing area (A8) should also be incorporated. As a common form of healing activity, drawing therapy has been shown to alleviate psychological anxiety in children and can serve as an effective auxiliary intervention during medical treatment (44). For the plant corner (A14), which is also classified as a One-dimensional need, the strict hygiene requirements of medical environments should be considered. Artificial plants, landscape boxes, or ornamental fish tanks are recommended as substitutes for real plants to reduce the risk of mosquito breeding and maintain sanitary conditions. Attractive needs refer to features that exceed user expectations and generate positive surprise. After ensuring the fulfillment of Must-be and One-dimensional needs, hospitals may selectively implement Attractive need projects according to their specific conditions and available resources. The construction of a sunroom (A15) in the infusion area and the projection of natural landscape imagery on walls (A13) reflect a strong desire among children and accompanying parents for contact with natural environments (45, 46). Research has demonstrated significant associations between light exposure, emotional states, and well-being (47–49), and patients in well-lit spaces have been reported to experience lower stress levels and slight reductions in pain perception (50). Medical space design should therefore emphasize the introduction of natural light and natural elements. Healthcare institutions with adequate conditions may also consider establishing a handicraft activity area (A9). Hands-on healing activities provide children with positive distraction during infusions and allow them to take home self-made crafts after treatment, reinforcing a sense of participation and achievement. In terms of art installations, dynamic and highly interactive forms (A7) are preferred, as they more effectively capture attention and enhance engagement within medical environments. AI interactive toys (A16), which combine technology with interactivity, further enrich children's experiences during prolonged infusion procedures. Previous research indicates that children associate ideal child-friendly hospitals with the availability of diverse toys and dedicated play spaces (51). Indifferent needs are those that exert minimal influence on satisfaction regardless of whether they are fulfilled. According to the results, static art installations (A6) and board game activity areas (A11) do not play a critical role in the infusion area and show limited effectiveness in improving children's emotional states during infusion. With respect to color preferences, the cool color scheme identified as a Must-be need should be prioritized, whereas warm color tones (A1) fall within the Indifferent category. It should also be noted that neutral tones (A3), classified as a Reverse need, should be avoided as the dominant color scheme in infusion spaces.

## 6 Limitations

This study is subject to several limitations. First, the sample size was relatively small, and data were collected from only two children's hospitals in Shanghai. Although the sample demonstrates a certain degree of representativeness, variations in geographic context, resource availability, and population characteristics across regions and institutions limit the direct generalizability of the findings to other settings. Future studies should expand the sample to include hospitals from diverse regions and at different institutional levels. Second, potential confounding factors, such as parental presence and interactions with medical staff, could not be fully controlled and may have influenced children's emotional responses. Third, the assessment of artistic preferences in infusion areas relied on a single methodological approach based solely on questionnaire surveys. Future research incorporating experimental designs, control groups, and investigations across multiple hospital spaces would strengthen the comprehensiveness and depth of the findings.

## 7 Conclusions

Based on a series of surveys, this study demonstrates that both children and parents strongly support the integration of art into hospital environments and recognize its necessity. The results also indicate that children's perception of existing hospital art remains limited and that different functional areas exert markedly different influences on children's emotional states. Focusing on pediatric infusion areas, the study further identified specific artistic needs and established their priority rankings. Overall, the findings provide empirical evidence supporting the incorporation of art into hospital environments, contributing to their evolution from treatment-oriented spaces to healing-oriented settings. The study offers a theoretical basis for the development of child-friendly medical spaces and provides practical recommendations for optimizing the design of pediatric infusion areas.

## Data Availability

The datasets presented in this article are not readily available because Since some of the original data were obtained from minors, the guardians do not wish for the information to be disclosed.. Requests to access the datasets should be directed to lywang@suse.edu.cn.
