# Supplementary material for: Support for art interventions in children's hospitals and design strategies for alleviating negative emotions
Source: Front Pediatr. 2026 Jan 12;13:1649799. doi: 10.3389/fped.2025.1649799 (PMC12833222; doi:10.3389/fped.2025.1649799)
Supplement: Supplementary file 1 [file Datasheet1.pdf]

Survey on Children's Perception of Hospital Art Atmosphere and Attitude towards Art Intervention in Hospitals

Hi, kids! We have some interesting questions for you, and we hope you can help answer them!

(1) Are you a boy or a girl?
- ○ Boy
- ○ Girl

(2) How old are you now?
- ○ 3-6 years old
- ○ 7-9 years old
- ○ 10-14 years old

(3) Have you noticed the wall paintings and artwork in hospitals?
- ○ Yeah, I' ve noticed them!
- ○ Not really.

(4) What do you think of these wall paintings and artwork?
- ○ Absolutely beautiful!
- ○ They're okay.
- ○ I don't really like them.

(5) Would you like to see more artworks in hospitals?
- ○ Definitely!
- ○ I don't mind either way.
- ○ Not really necessary.

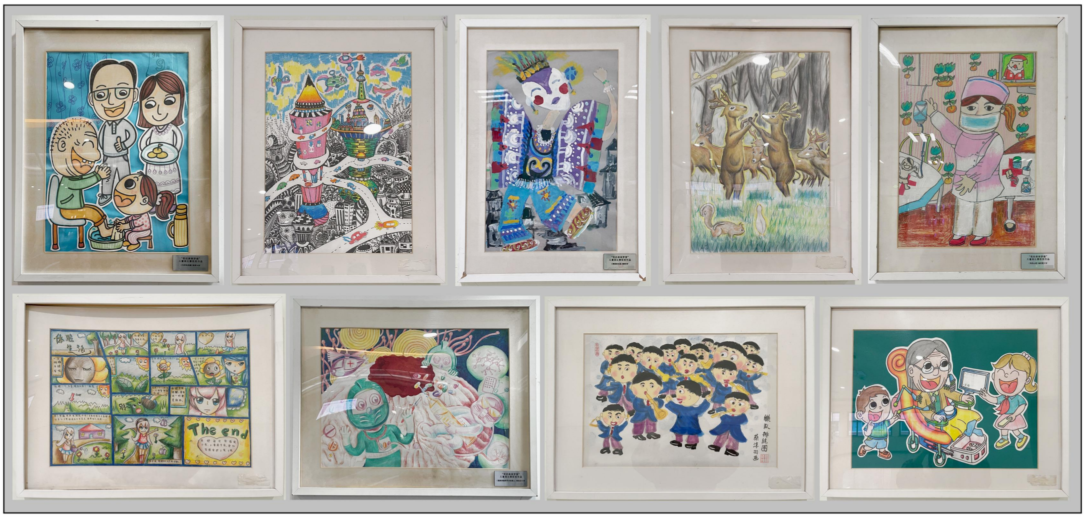

Survey on Parents' Attitudes towards Art Intervention

Dear Parent,

Hello! We are currently conducting a study on the optimization of public spaces in children's hospitals, with the goal of improving the medical experience and emotional well-being of young patients through art intervention. This survey is anonymous, and the data collected will be used solely for academic research purposes. Thank you for your support!

(1) Does the artwork positively influence your child's mood during their hospital visit?

○ Strongly agree
○ Agree
○ Neutral
○ Disagree
○ Strongly disagree

(2) Do you support the incorporation of additional artworks or therapeutic activities in pediatric healthcare facilities?

○ Strongly support
○ Support
○ Neutral
○ Somewhat oppose
○ Strongly oppose

(3) Do you support the creation of designated art exhibition spaces within children's hospitals?

○ Strongly support
○ Support
○ Neutral
○ Somewhat oppose
○ Strongly oppose

Thank you for your participation! Your feedback will provide important support for improving children's medical experience. Thank you!

Survey on the Demand for Art and Healing Activity Zones in Children' s Hospital Infusion Areas

Hi there, parent/friend!
We' re working on a super cool study about making the infusion area in children' s hospitals way more fun and comforting through art and healing activities. Here' s the list of needs for this study. Please read each description and imagine how you' d feel if the infusion area actually had this feature, or if it didn' t have it at all. Take your time and mark "√" in each "○." Be honest with your answers—I really appreciate your input! All the information will be kept super safe and used only for research. Thank you! You' re awesome!

1. **Attitude towards warm-colored space**	
The space is warm-toned	The space is not warm-toned
○ I like it<br>○ I expect it<br>○ I am neutral<br>○ I can tolerate it<br>○ I dislike it	○ I like it<br>○ I expect it<br>○ I am neutral<br>○ I can tolerate it<br>○ I dislike it

2. **Attitude towards cool-colored space**	
The space is cool-toned	The space is not cool-toned
○ I like it<br>○ I expect it<br>○ I am neutral<br>○ I can tolerate it<br>○ I dislike it	○ I like it<br>○ I expect it<br>○ I am neutral<br>○ I can tolerate it<br>○ I dislike it

3. **Attitude towards neutral-colored space**	
The space is neutral-toned	The space is not neutral-toned
○ I like it<br>○ I expect it<br>○ I am neutral<br>○ I can tolerate it<br>○ I dislike it	○ I like it<br>○ I expect it<br>○ I am neutral<br>○ I can tolerate it<br>○ I dislike it

4. **Attitude towards wall murals**	
There are murals on the walls	There are no murals on the walls
○ I like it<br>○ I expect it<br>○ I am neutral<br>○ I can tolerate it<br>○ I dislike it	○ I like it<br>○ I expect it<br>○ I am neutral<br>○ I can tolerate it<br>○ I dislike it

5. **Attitude towards art paintings**	
Art paintings are hung on the walls	Art paintings are not hung on the walls
○ I like it	○ I like it
○ I expect it	○ I expect it
○ I am neutral	○ I am neutral
○ I can tolerate it	○ I can tolerate it
○ I dislike it	○ I dislike it

6. **Attitude towards static art sculptures/installations**	
Static art sculptures are placed	Static art sculptures are not placed
○ I like it	○ I like it
○ I expect it	○ I expect it
○ I am neutral	○ I am neutral
○ I can tolerate it	○ I can tolerate it
○ I dislike it	○ I dislike it

7. **Attitude towards interactive art installations**	
Interactive art installations are placed	Interactive art installations are not placed
○ I like it	○ I like it
○ I expect it	○ I expect it
○ I am neutral	○ I am neutral
○ I can tolerate it	○ I can tolerate it
○ I dislike it	○ I dislike it

8. **Attitude towards drawing areas**	
A drawing area is set up	A drawing area is not set up
○ I like it	○ I like it
○ I expect it	○ I expect it
○ I am neutral	○ I am neutral
○ I can tolerate it	○ I can tolerate it
○ I dislike it	○ I dislike it

9. **Attitude towards handicraft-making areas**	
A handicraft area is set up	A handicraft area is not set up
○ I like it	○ I like it
○ I expect it	○ I expect it
○ I am neutral	○ I am neutral
○ I can tolerate it	○ I can tolerate it
○ I dislike it	○ I dislike it

10. **Attitude towards book corners**	
A reading corner is set up	A reading corner is not set up
○ I like it	○ I like it
○ I expect it	○ I expect it
○ I am neutral	○ I am neutral
○ I can tolerate it	○ I can tolerate it
○ I dislike it	○ I dislike it

11. **Attitude towards roard Game areas**	
A chess area is set up	A chess area is not set up
○ I like it	○ I like it
○ I expect it	○ I expect it
○ I am neutral	○ I am neutral
○ I can tolerate it	○ I can tolerate it
○ I dislike it	○ I dislike it

12. **Attitude towards movie-watching areas**	
A viewing area is set up	A viewing area is not set up
○ I like it	○ I like it
○ I expect it	○ I expect it
○ I am neutral	○ I am neutral
○ I can tolerate it	○ I can tolerate it
○ I dislike it	○ I dislike it

13. **Attitude towards wall projections of natural themes**	
Nature-themed images are projected on the walls	Nature-themed images are not projected on the walls
○ I like it	○ I like it
○ I expect it	○ I expect it
○ I am neutral	○ I am neutral
○ I can tolerate it	○ I can tolerate it
○ I dislike it	○ I dislike it

14. **Attitude towards artificial plant corners**	
A plant corner is set up	A plant corner is not set up
○ I like it	○ I like it
○ I expect it	○ I expect it
○ I am neutral	○ I am neutral
○ I can tolerate it	○ I can tolerate it
○ I dislike it	○ I dislike it

15. **Attitude towards simulated sunlight (sunroom)**	
A simulated sunlight area (sunroom) is set up	A simulated sunlight area (sunroom) is not set up
○ I like it	○ I like it
○ I expect it	○ I expect it
○ I am neutral	○ I am neutral
○ I can tolerate it	○ I can tolerate it
○ I dislike it	○ I dislike it

16. **Attitude towards artificial intelligence (AI) interactive dolls**	
AI interactive dolls are set up	AI interactive dolls are not set up
○ I like it	○ I like it
○ I expect it	○ I expect it
○ I am neutral	○ I am neutral
○ I can tolerate it	○ I can tolerate it
○ I dislike it	○ I dislike it

Thank you so much!
You're incredible! Wishing you a healthy body and a happy heart every day!
